# Environmental Stability and Its Importance for the Emergence of Darwinian Evolution

**DOI:** 10.3390/life13101960

**Published:** 2023-09-25

**Authors:** Khushi R. Daga, Mensura Feray Çoşar, Abigail Lowenkron, Jihua Hao, Joti Rouillard

**Affiliations:** 1 Blue Marble Space Institute of Science, Seattle, WA 98104, USA; khushirdaga@gmail.com (K.R.D.); cosar18@itu.edu.tr (M.F.Ç.); arlowenk@eckerd.edu (A.L.); 2 Deep Space Exploration Laboratory/CAS Key Laboratory of Crust-Mantle Materials and Environments, University of Science and Technology of China, Hefei 230026, China

**Keywords:** early evolution, hydrothermal environments, protocells, population dynamics, numerical modelling

## Abstract

The emergence of Darwinian evolution represents a central point in the history of life as we know it. However, it is generally assumed that the environments in which life appeared were hydrothermal environments, with highly variable conditions in terms of pH, temperature or redox levels. Are evolutionary processes favored to appear in such settings, where the target of biological adaptation changes over time? How would the first evolving populations compete with non-evolving populations? Using a numerical model, we explore the effect of environmental variation on the outcome of the competition between evolving and non-evolving populations of protocells. Our study found that, while evolving protocells consistently outcompete non-evolving populations in stable environments, they are outcompeted in variable environments when environmental variations occur on a timescale similar to the average duration of a generation. This is due to the energetic burden represented by adaptation to the wrong environmental conditions. Since the timescale of temperature variation in natural hydrothermal settings overlaps with the average prokaryote generation time, the current work indicates that a solution must have been found by early life to overcome this threshold.

## 1. Introduction

Beyond the answers provided by mythology and religion, the question of how life originated on Earth has fascinated scientists for well over a century [1]. Since then, scientists have defined cells as the basic unit of life on Earth. Thus, questioning the origins of life has mainly meant investigating how the first cells came to be. Because cells are understood as being an integrated network of functional units (a compartment, a genome, and a metabolism), the main research axes explored in the origins of life field concern the origins of these different units: the first genomic material [2,3,4], the emergence of metabolism [5,6,7,8,9,10,11], and the first compartments [12,13,14,15,16,17,18,19,20].

The emergence of evolutionary processes is also a critical prerequisite for cellular life to have reached the complexity and diversity it shows today. Several hypotheses have been put forward on the origin of evolution, and while the appearance of nucleic acids is critical, evolution may have occurred before the genome existed [21,22,23,24,25].

Yet, when hypothesizing about the emergence and evolution of life on Earth, it must be remembered that not the entire space of possibility is relevant. Rather, a proposed scenario must remain consistent with the geological environment of its time [26], as inferred on the basis of modern environments and Early Earth paleoenvironmental records.

The two key environmental contenders for the origins of early life are oceanic hydrothermal vents and hot springs [5,27,28,29,30,31,32]. These environments exhibit unique spatiotemporal variations [33,34,35]. Would these fluctuating conditions favor or inhibit evolution? Previous research has emphasized the potential of spatial variations for the evolution of life [17,25,36]. However, a population evolving to be more efficient under certain environmental conditions may be detrimental to long-term population survival, because the environment changes over time. Indeed, Darwinian adaptation (i.e., a combination of heritable variation and selection) to certain conditions could be accompanied by decreased efficiency under other conditions. This means that there is an evolutionary trade-off between short-term and long-term population survival in unstable environments. As far as we are aware, these key aspects have not yet been developed in the literature on Early Life, and they will therefore form the basis for the current study.

Here, using a numerical model, we aim to determine the conditions of environmental stability under which evolving protocells would have a long-term survival advantage over non-evolving protocells.

## 2. Presentation of the Model

In the following, parameters written in italics change during the course of the simulation, and parameters written in bold are constant for the duration of the simulation. The numerical model describes a population of *N* protocells inside a compartment containing five different types of molecule: food molecules (concentration *F*), and growth catalyst molecules A_1_, …, A_4_ (constant concentrations in the compartment **A_X,C_**). This compartment could represent pores in hydrothermal chimneys, or pools in terrestrial fields of hot springs. Neither protocells nor molecules A_1_, …, A_4_ are able to enter or exit the compartment, but there is a regular input of food molecules at the rate **Fi**. The conditions in the compartment can also change. The environmental parameter *P* (which represents temperature, pH, redox level, etc.) varies following a sinusoidal function of period **Pt** and amplitude **Pa**. Protocells ingest food, to achieve their own maintenance and growth. Once the volume of a protocell reaches a threshold value (equal to double their initial volume), it divides into two daughter cells.

It is assumed that food enters the cells through diffusion, following Fick’s law [37]. Since all food that enters the cells is consumed immediately, the amount of food entering a cell during an infinitesimal amount of time dt is equal to:

*df* = **Df**·*V*^(2/3)^·dt,

where *V* is the cell volume and **Df** is the diffusion constant.

**Figure 1:**
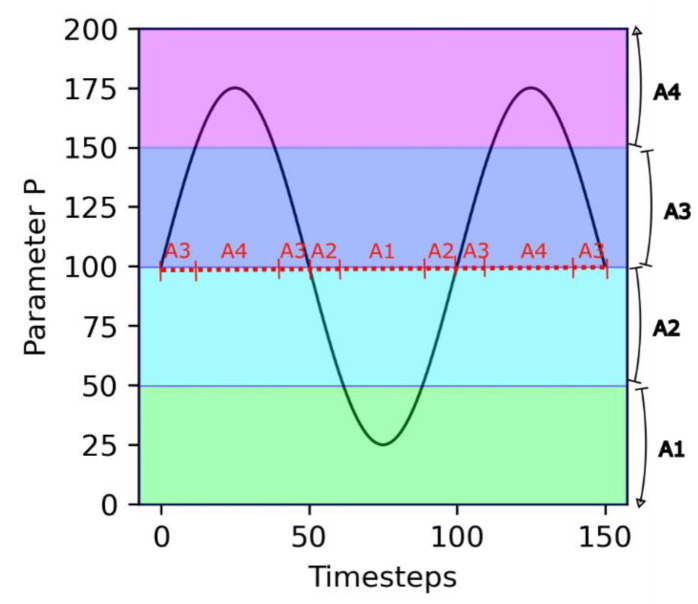
Principle of environmental dependency. Parameter *P* varies following a sinusoidal trend. The different growth catalyst molecules are each active for a specific range of values of *P* (shaded areas). As a consequence, over time, there are transitions in the nature of the active growth catalyst molecule (red dashed line).

An important assumption in this model is that the dependence on the environment is mediated by the growth catalyst molecules A1, …, A4. Depending on the value of the environmental parameter *P*, different molecules are active for growth catalysis (see Figure 1). This aspect simulates the effect of environmental parameters, such as temperature or pH, on the speciation of critical biomolecules.

Catalyst molecules are exchanged between the cell and the compartment through diffusion, but they can also be actively uptaken by cells (e.g., through energy consumption). This active uptake can be represented by protein membrane pumps. The change in concentration in the cell of molecule A_x_, *A_X_*, during an infinitesimal amount of time is therefore:

d*A_X_* = ((**D**·(**A_X,C_** − *A_X_*) + *Ux*)·dt)/*V*^(1/3)^

where **D** is the constant of diffusion for catalyst molecules, **A_X,C_** is the concentration of A_x_ in the compartment, and *Ux* is the active uptake rate of Ax.

The food required for maintenance and for the active uptake of molecules A_1_, …, A_4_ during dt is:

*dfm* = (**fm**·*V* + **fu** ∑ *Ux*)·dt

where **fm** and **fu** are two constants.

Any remaining food is used for the growth of the protocell; the volume change d*V* during dt is therefore:

d*V* = **Gr**·(*A_i_*)^2^·(d*f* − d*fm*)

where the growth rate **Gr** is a constant and *A_i_* is the concentration in the growth catalyst that is active under the current value of *P*.

The concentrations of the molecules *A_X_* in the protocells are constantly adjusted in response to changes in protocell volume *V*, in accordance with the following equation:

dA_X_/A_X_ = V/dV

Protocells may die with a probability that increases when the food ‘debt’ *dfm* − *df* increases. This represents the necessity for cells to spend energy to maintain their structure. The probability of cell death as a function of *dfm* − *df* follows a sigmoidal law (see Figure S1), with a threshold for *dfm* − *df* = 0, according to:

Probability(death during dt) = (1/2)·(1 − 1/(1 + exp(−**L**·(*dfm* − *df*))))

where **L** is a constant.

When a protocell divides, the daughter cells have the same concentration of *A_X_* and a volume corresponding to half of the volume of the mother cell. For non-evolving protocells, the rates of active uptake *U_x_* stay the same between the mother and daughter cells. For evolving protocells, the rates of active uptake *U_x_* change stochastically in the new generation. The distribution of changes in *U_x_* (Δ*U_x_*) between generations follows a normal law:

Probability(Δ*U_x_* = i) = (1/**S**·(2π) ^(1/2)^)·exp((−i^2^)/2·**S**^2^)

where **S** is a constant; higher values of **S** translate into faster changes in *U_x_* between generations.

## 3. Results

### 3.1. Reference Population

First, we describe how a population of non-evolving protocells (also referred to as ‘Type 1’) behaves under the reference conditions defined below:

**F0** = 0.5 and **N0** = 100 are the initial food concentration and initial number of protocells in the compartment, respectively.

**Fi** = 0.2 is the rate of food input in the compartment.

**A_X,C_** = 100 refers to the concentration of catalyst molecules in the compartment and the protocell.

Under these reference conditions, since the cells do not evolve, the environmental parameter *P* does not influence the concentrations *A_1_*, …, *A_4_*. The outcome of the model is therefore unaffected by *P*.

The reference parameters of the protocells are: initial cell volume **V0** = 1, constant active uptake rate *U_x_* = 10 for each catalyst molecule, **Df** = 0.01, **D** = 0.1, **fm** = 0.001, **fu** = 1·10 ^(−5)^, **Gr** = 0.005, and **L** = 1000.

**Figure 2:**
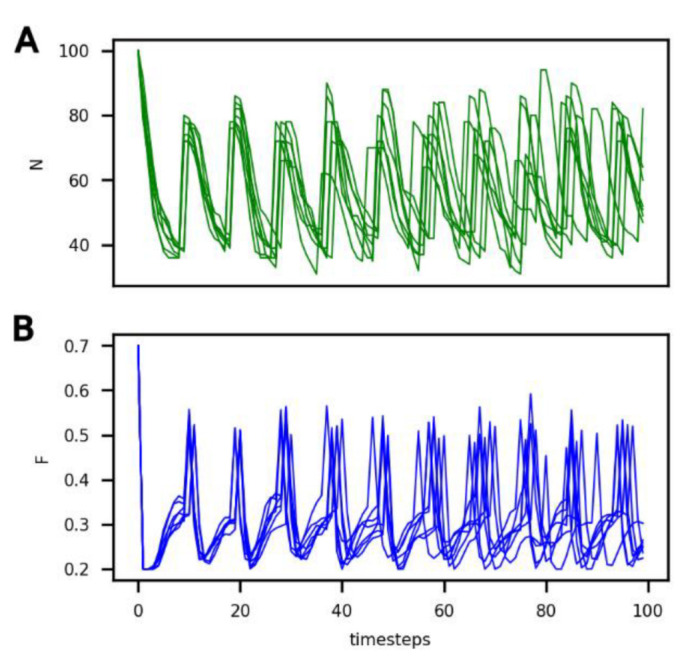
Evolution of (**A**) the number of non-evolving protocells (*N*, green curve) and of (**B**) the food concentration in the compartment (*F*, blue curve) over the course of the simulation under the reference conditions and for 6 different iterations.

In Figure 2, it can be seen that the number of cells (*N*) and the amount of food in the compartment (*F*) vary over time under these reference conditions for six different iterations. Variation between individual runs under these conditions is solely due to the randomness of protocell death (see the probability law for death described in Section 2).

It can be seen that, under these conditions, the system evolves towards a dynamic equilibrium, characterized by:

(1) A common period of oscillations for *N* and *F* of approximately 10 timesteps, which can also be considered an average generation time;
(2) A number of protocells in the compartment oscillating between 40 and 80; and
(3) An amount of food in the compartment oscillating between 0.2 and 0.5.

**Figure 3:**
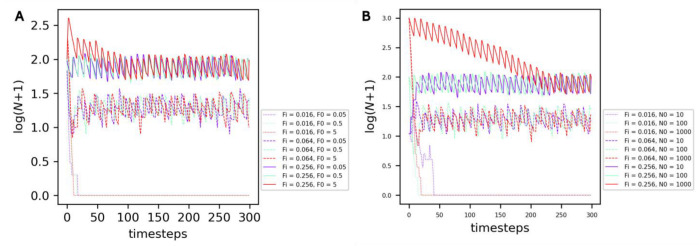
Evolution of the number of non-evolving protocells over the course of the simulation under the reference conditions. (**A**) Comparison of different combinations of food input rates **Fi** (curve style) and initial food amounts **F0** (curve color). (**B**) Comparison of different combinations of food input rates **Fi** (curve style) and initial cell numbers **N0** (curve color).

The average number of protocells reached under equilibrium—which can be named the ‘carrying capacity’ of the compartment—is not dependent on the initial amount of food **F0** (Figure 3A, curve color) or on the initial number of cells **N0** (Figure 3B, curve color) in the compartment, but depends on the rate of food input in the compartment **F**i (Figure 3, curve style).

### 3.2. Influence of Protocell Constants

We then explore how the different constants that are intrinsic to protocells, **Df**, **fm**, **Gr** and **L**, influence the protocell populations.

**Figure 4:**
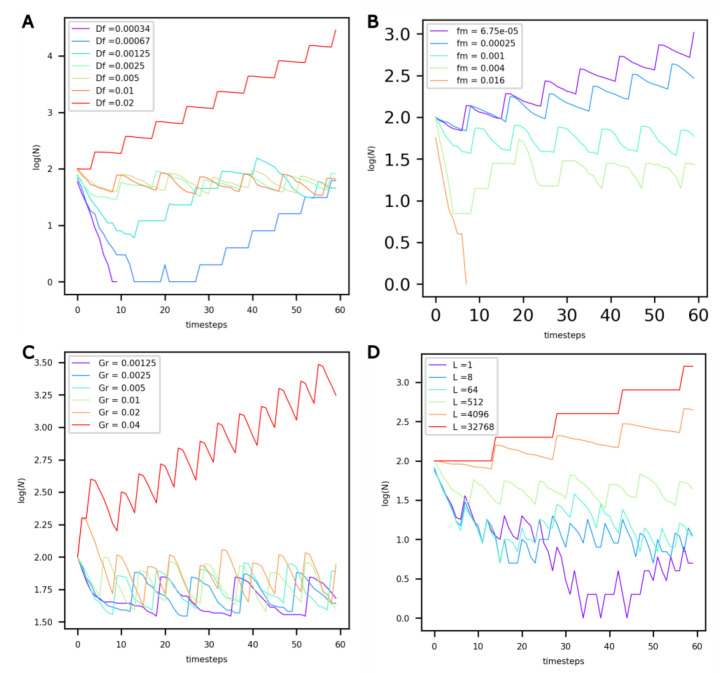
Evolution of the number of non-evolving protocells (vertical axis, log-scaled) over the course of the simulation under different values of (**A**) food diffusion factor **D**f, (**B**) factor of maintenance **fm**, (**C**) volume growth factor **Gr**, and (**D**) life factor **L**.

It can be observed that for low **Df** values (Figure 4A, **Df** < 0.00125), the extremely slow diffusion of food in the protocells prevents their maintenance (see purple curve). For higher values, and up to **Df** = 0.01, the value of **Df** does not influence the population dynamics—they reach the same dynamic equilibrium. However, for values beyond **Df** = 0.01, the number of protocells in the compartment appears to grow exponentially.

Decreasing the food maintenance factor **fm** has a marked influence on population dynamics (Figure 4B). Lower values of **fm** translate into a lower probability of death and more food available for growth. For values of **fm** less than 0.00025, the number of protocells in the compartment increases exponentially. On the other hand, for **fm** = 0.016, the maintenance cost is too high, and the population dies out.

An increase in the growth factor **Gr** (Figure 4C) primarily modifies the period of oscillations, or generation time, of the system. With increasing **Gr**, growth is accelerated, and the period of oscillations decreases. Once Gr reaches a threshold value, the dynamic equilibrium is altered, and an exponential increase in the number of protocells is observed.

When the life factor **L** increases (Figure 4D), the probability of death of protocells decreases. For **L** values equal to 4000 or higher, the probability of death is very low, and the number of protocells increases exponentially.

Due to obvious resource limitations, the exponential growth of a population in a closed compartment cannot be realistically sustained over extended periods of time. Consequently, when modeling protocell populations in hydrothermal compartments—whether those compartments represent chimney pores or larger hot spring pools—the results shown in Figure 4 indicate that upper boundaries of 0.1, 0.001, 0.02 and 1000 must be adopted for **Df**, **fm**, **Gr** and **L**, respectively.

### 3.3. Competition between Evolving and Non-Evolving Population—Stable Environment

**Figure 5:**
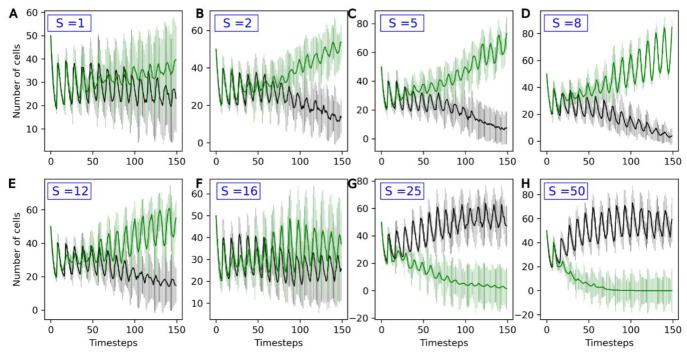
Number of non-evolving protocells (black curve) and number of evolving protocells (green curve) plotted over the course of the simulation. From top to bottom and left to right, the eight graphs present increasingly high values for the evolution rate **S:** (**A**) **S** = 1, (**B**) **S** = 2, (**C**) **S** = 5, (**D**) **S** = 8, (**E**) **S** = 12, (**F**) **S** = 16, (**G**) **S** = 25, (**H**) **S** = 50. The average and standard deviation, calculated over 15 iterations, are presented.

The parameters used in the following are the reference parameters listed in Section 3.1. A second population of protocells—the evolving population, also referred to as ‘Type 2’—is added to the compartment. For this population, the active uptake rates *U_1_*, …, *U_4_* of the four catalyst molecules A_1_, …, A_4_ vary stochastically between generations. The average change in *U_x_* between two timesteps is **S**, in parallel to a non-genomic evolution rate. Figure 5 shows how the two populations, evolving and non-evolving, compete with each other for different evolution rates **S** of the Type 2 population.

For low values of **S** (**S** = 1), the outcome of competition is highly variable. On average, evolving populations tend to outcompete non-evolving ones, but the standard deviations for both curves strongly overlap.

**Figure 6:**
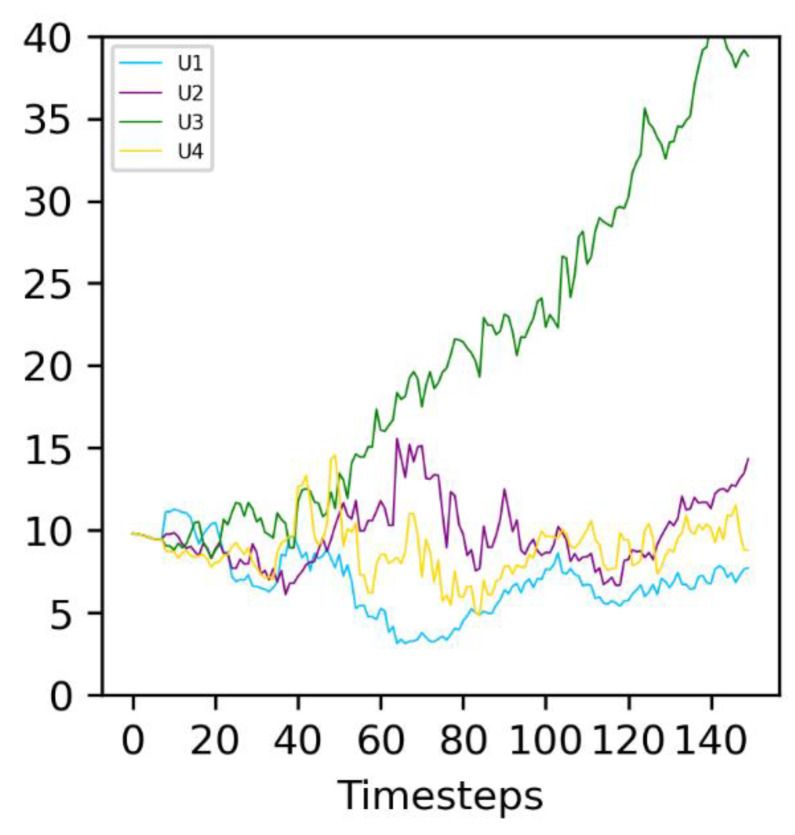
Evolution of the average of the four different U_x_ in evolving (Type 2) protocells in an example run. The environmental parameter is constant at *P* = 100, corresponding to molecule A_3_ being active (see Figure 1). The correspondence between the curve color and U_x_ is given on the graph legend. The average values of U_x_, calculated over all Type 2 protocells in the simulation, are represented.

For **S** values up to 8, increasing **S** is favorable to evolving populations (green curves), which more consistently outcompete the non-evolving ones. In this stable environment, the same catalyst molecule A_i_ is active for the entire simulation. Type 2 protocells that randomly see an increase in the active intake for this molecule, *U_i_*, grow and divide faster than other protocells: they are positively selected. This can be observed in Figure 6: the molecule A_3_ is the active catalyst under these conditions, and the average *U_3_* (green line) in Type 2 protocells increases throughout the duration of the run. In general, the Type 2 population therefore adapts to this environment, leading to it outcompeting the non-evolving Type 1 population.

However, beyond **S** = 8, there is a reversal in this trend, and the outcome of the competition varies between runs, even reversing in favor of the Type 1 population for **S** = 16 and higher (Figure 5F–H). This is because, with high evolution rates, although an increase in *U_x_* in one generation will lead to a higher growth rate, this increase is likely to be compensated by a decrease in the following generation. As a consequence, *U_x_* and the growth rate are very weakly correlated across generations. This is a phenomenon akin to the error threshold described in evolutionary biology [38], and prevents the adaptation of the Type 2 protocell population to its environment.

### 3.4. Competition between Evolving and Non-Evolving Populations—Changing Environment

In the following, the reference values used in Section 3.1 are employed, but sinusoidal temporal variations in the environmental parameter *P* (see Figure 1) are introduced. We studied how these *P* variations influence the outcome of the competition between non-evolving and evolving protocells. In order to discard the known effect of the error threshold, a maximum **S** value of 12 was used in these simulations.

**Figure 7:**
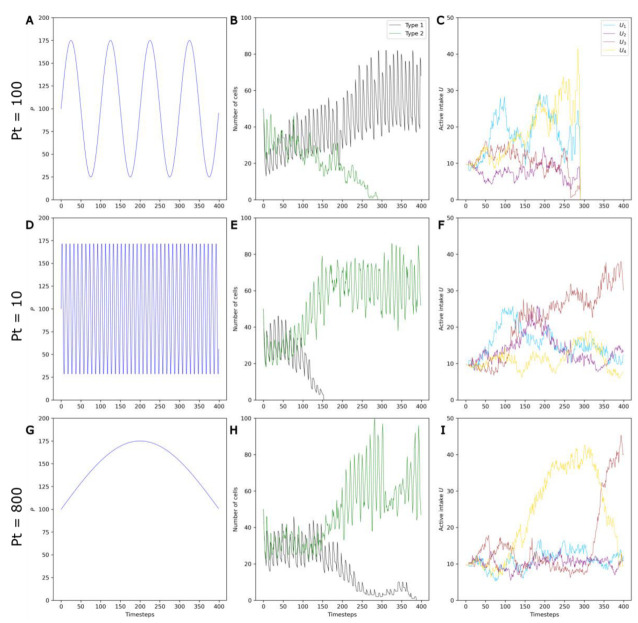
Example runs with a **Pa** of 75 and **S** of 8 illustrating the effect of **Pt** on selection and on competition. Three different values of **Pt** (**Pt** = 100, **Pt** = 10 and **Pt** = 800) are shown on the three rows. (**A**,**D**,**G**) *P* variations. (**B**,**E**,**H**) Changes in the numbers of non-evolving protocells (black curve) and evolving protocells (green curve) over the course of the simulation. (**C**,**F**,**I**) Change in the average active intake for the four molecules A_1_, …, A_4_ in Type 2 protocells over the course of the simulation.

Three example runs with **Pa** = 75, **S** = 8 and three values of **Pt**—100, 10 and 800—are shown Figure 7.

First, it can be clearly observed that, in contrast to stable environments, evolving protocells do not always outcompete non-evolving protocells in changing environments (Figure 7A,B). This is in accordance with the initial hypothesis that environmental variations can be detrimental to evolving protocells. During a period in which the molecule A_i_ is the active catalyst, evolving protocells with higher *U_i_*—that is, higher active intake for the molecule A_i_—are selected (see Figure 5). However, as *P* oscillates and the active catalyst changes to molecule A_j_ (Figure S1), this higher *U_i_* becomes a nonfunctional energetic burden.

Second, the frequency of environmental variations also appears to influence the system, with evolving populations that are more favored when fluctuations are very fast or very slow (Figure 7E–H).

**Figure 8:**
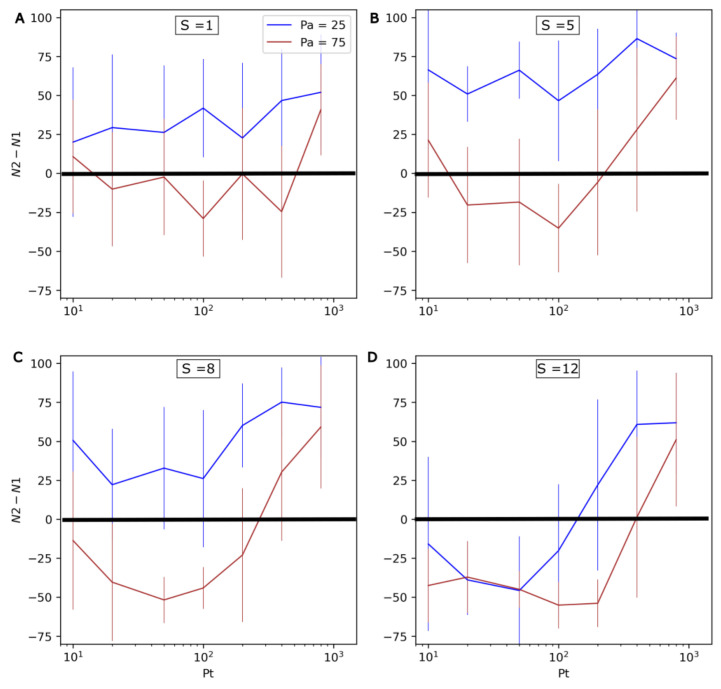
Influence of **Pa, Pt** and **S** on the competition between non-evolving (Type 1) and evolving (Type 2) protocells. From top to bottom and left to right, the four graphs present increasingly high values for the evolution rate **S:** (**A**) **S** = 1, (**B**) **S** = 5, (**C**) **S** = 8, (**D**) **S** = 12. The differences in numbers of both types of protocell at the end the run (*N2-N1*) are shown on the vertical axis (the average and standard deviation, calculated over 20 iterations of 400 timesteps, are presented). As a consequence, positive values mean that evolving protocells outcompete non-evolving protocells, while negative values mean that the former are outcompeted by the latter. The horizontal axis represents the period of **P** oscillations, **Pt**, and the colors of the curves correspond to two different amplitudes of **P** (**Pa** = 25 in blue, and **Pa** = 75 in red).

In order to more systematically assess the effect of environmental oscillations, different combinations of values for the evolution rate **S** (1 to 12), the period of environmental oscillations **Pt** (10 to 800) and their amplitude **Pa** (25 or 75) were explored. For each combination of values, 20 simulation iterations of 400 timesteps were conducted. The results are shown in Figure 8. *N1* and *N2* respectively refer to the number of non-evolving (Type 1) and evolving (Type 2) protocells at the end of the simulations. Here, the standard deviation is important, and highlights that although general trends are clear, there can be large differences between individual runs.

The possibility of evolving protocells being outcompeted in changing environments is confirmed by the negative *N2-N1* values observed in Figure 8. Additionally, as the amplitude of *P* oscillations (**Pa**) increases, it can be observed that evolving protocells generally become less competitive (the brown curve is significantly lower than the blue curve).

In accordance with the runs presented in Figure 7, the rate of environmental change is found to have a nonlinear influence on the system. Increasing **Pt** from 10 to ~100 negatively impacts Type 2 protocells. However, a further increase in **Pt** from ~100 to 800 favors Type 2 protocells. This effect appears even more significant at high mutation rates **S** and/or with greater environmental changes (higher **Pa**).

This effect of **Pt** can be understood in light of the average doubling time of protocells, which is roughly 10–15 timesteps with the parameters used here (Figure 2, Figure 3, Figure 5 and Figure 8).

For short periods (**P_T_** ~ 10), the transition between active catalysts occurs more often than protocell division, so selection does not occur under the same environmental conditions over several generations, limiting the development of energetic burdens.

For intermediate periods (**Pt** ~ 100), selection occurs with the same active catalyst over a few generations. However, the frequent change in the active catalyst leads to frequent energetic burdens.

For longer periods (**Pt** ~ 800), selection occurs under the same active catalyst for a large number of generations. In Figure 7I, the successive selection of *U_3_*, *U_4_*, and *U_3_* again can be clearly seen. Consequently, the energetic burdens are larger (note the significant drop in Type 2 cells at the timesteps 100 and 300), but also less frequent.

Both the degree and frequency of energetic burdens thus play a role in the long-term competitive abilities of evolving protocells.

## 4. Discussion

### 4.1. Comparison of the Model with Hydrothermal Environments and Single-Celled Organisms

One important result is that the effect of environmental variations depends on their time scale relative to protocell generation period (Figure 8). The current model indicates that environmental variations that are shorter or much longer than the generation period do not negatively affect evolving protocells. However, environmental variations that are only slightly longer than the generation time disfavor evolving protocells. In the following, we compare timescales of (1) environmental variations in modern hydrothermal environments and of (2) generation or doubling times in prokaryote cells.

The existence of spatiotemporal variations in parameters such as temperature, pH and redox levels in hydrothermal environments are well known, but, partly due to technical difficulties, few studies have reported time series. Nonetheless, precise temperature measurements from several marine hydrothermal vents ([33] and references therein) indicate short-term temperature variations on the order of 0.1 K/min, with longer-term variations on the order of 30 K/day. Time series from the siliceous hot spring of El Tatio, Chile [35] and from Fox Glacier, Uruni and Hanmer Springs, New Zealand [33] indicate daily variations on the order of 10 K, partly due to daily fluctuations in air temperature. Volcanic fumaroles from the La Soufriere volcano were found to have several orders of temperature variations, with daily variations of 1–2 K and monthly variations of 10 K [39].

**Figure 9:**
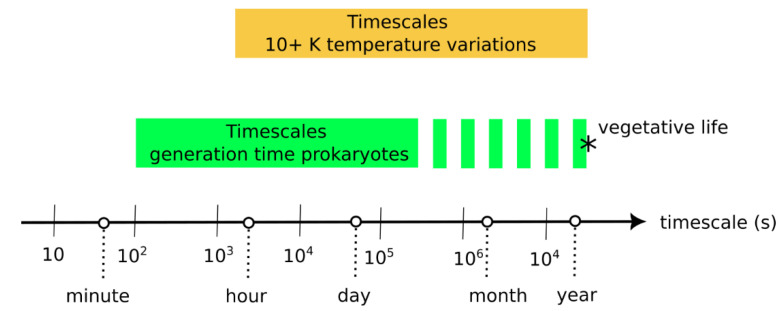
Comparison of (1) the timescales of environmental variation in hydrothermal environments—where temperature variations of more than 10 K are considered—against (2) the average timescales of generation/doubling times in prokaryote populations [33,34,35,40,41,42,44]. The asterisk (*) represents the uncertainty of the position of the upper boundary for the generation times, which is little known for vegetative life (e.g., in the Deep Biosphere).

On the other hand, laboratory cultivation experiments indicate typical generation times for prokaryotes ranging from a few minutes to several days [40,41,42,43]. Dormant or vegetative cells, in the deep biosphere, for example, probably present much longer doubling times on the order of a year [44]. The generation timescale of prokaryotes and the environmental variation timescales therefore significantly overlap (Figure 9). Note that protocell division has also been achieved in the laboratory, with a combined time for growth and division ranging from ~30 min [45] to a few days [46], which are similar durations to prokaryote generation time.

Assuming that these timescales are similar to those existing when early life evolved, this overlap indicates that environmental variation had a probable influence on the survival and the competitive abilities of the first evolving protocells.

The current study also indicates that evolution rate is an important factor for the outcome of the model. In stable environments, increasing **S** from 1 to 8 leads to evolving protocells outcompeting non-evolving protocells in a shorter time (Figure 5). A further increase leads to error thresholds, disfavoring evolving protocells, which are outcompeted for **S** values of 16 and above. In a changing environment, increasing **S** from 1 to 5 favors evolving protocells, but a further increase is detrimental to them (Figure 8).

In modern prokaryotic cells, mutations occur with a frequency of 1/10^9^ base pairs per generation in DNA [47], and with a frequency of around 1/10^5^ bases during RNA_m_ transcription [48,49]. However, the evolution rate used in the current model is a phenotypic rate, which describes the average change of active uptake *U_x_*. It is very difficult to compare those phenotypic rates with mutation rates, since the effect of genome changes on phenotype is not linear and highly variable depending on the phenotypic parameter. Relative to the reference values of *U_x_* of 100, **S** rates of 1–12 correspond to changes of 1–12% per generation, which seems high. However, homeostatic processes in protocells were probably much simpler than they are today, potentially leading to higher phenotypic plasticity.

The current study indicates that heritable changes in the first evolving protocells could not have exceeded the error threshold (**S** ~ 16 here), since this would have (i) prevented any adaptation to the environment and (ii) led to evolving populations being systematically outcompeted by non-evolving populations (Figure 5). Alternatively, with low evolution rates (**S** = 1 here), the outcome of competition between evolving and non-evolving populations is variable in both stable (Figure 5) and variable environments (Figure 8). Two alternative scenarios can therefore be proposed: either (1) the first evolving protocells had intermediary evolution rates, corresponding to **S** ~ 2–12, or (2) the first evolving protocells evolved slowly, with **S** values of 1 or lower, but competition with non-evolving protocells occurred several times, maybe in several places, leading evolving protocells to outcompete in at least one occurrence.

### 4.2. Limitations of the Model

This model is a strong simplification of the biology of protocells. First, it is assumed that evolution relies on the selection of stochastic changes in the active uptake of different catalyst molecules. In line with previous studies [21,22,23,24,25], we consider it unlikely that the first evolutionary processes were based on genomes. However, this is only one representation, and early evolution may have been vastly different from what is modeled here e.g., [24].

It is also assumed that the environmental parameter *P* influences protocells by controlling the nature of the active catalyst molecule. This type of environmental influence is consistent with observations in modern environments, with temperature, pH or redox levels affecting the speciation of critical biomolecules. However, the environment affects biochemistry and metabolism in a much more complex way. In today’s cells, for example, temperature or pH are known to modify the conformation of proteins, often leading to their inactivation. We are limited in this modeling work by contemporary knowledge of early life and of the biochemical functioning of protocells.

Lastly, in this model, catalyst uptake consumes protocells’ energy, meaning that changes in the nature of the active catalyst can lead to energetic burdens for evolving protocells. Such a phenomenon is unlikely in modern cells, where the characteristics of cytoplasm are regulated through homeostasis. However, in protocells, homeostatic processes were likely less developed, and the active uptake represents the (risky) energetic investment required for adaptation.

In order to give further breadth to these results, the model could be improved to test (1) alternative modalities of evolution, (2) alternative modalities of coupling between the environment and protocells, and (3) alternative modalities representing the energetic investment of adaptation.

## 5. Conclusions

The emergence of the first evolutionary processes on Early Earth remains a fundamental step in the development of life as we know it. The current study sheds some critical light on the conditions necessary for the first evolving protocells to survive over time in their environment.

In this study, using a numerical model, we assessed the influence of various factors on the outcome of the competition between the first evolving protocells and non-evolving protocells.

It was found that, through adaptation, in stable environments, evolving protocells with small to moderate evolution rates can consistently outcompete non-evolving protocells in a few generations. However, very high rates of evolution prevent adaptation and lead to the demise of evolving protocells.

With this model, we also confirm the hypothesis that in environments with fluctuating conditions, such as hydrothermal environments, evolving protocells can be outcompeted by non-evolving ones. This is because adaptation to certain conditions requires an energetic investment that becomes a burden if conditions change. This phenomenon is amplified when the environmental changes are greater (which is modeled here by the transition between a larger number of catalyst molecules).

The period of environmental change is also critical, since evolving protocells are only negatively affected by changes that occur on timescales greater than one generation and shorter than tens of generations—intermediate timescales that actually also correspond to the timescales of temperature variations in modern hydrothermal environments.

Lastly, during this study we noted large variations between individual runs (see error bars in Figure 8). Consequently, even if evolving populations are outcompeted on average, where evolutionary processes appear a large number of times and/or in many places, they may survive in some instances.

Overall, this study emphasizes the need to consider more thoroughly in future studies how early protocells interacted with their complex biotic and abiotic environment on the origin of life.

## Data Availability

Source code is available as a Supplementary Materials.

## Supplementary Materials

The following supporting information can be downloaded at: https://www.mdpi.com/article/10.3390/life13101960/s1, Figure S1: Probability of protocell death depending on food debt; Numerical model code: script of the model.

## Nomenclature

*N*	Number of protocells in the compartment (no unit)
**N0**	Initial number of cells
*F*	Concentration in food molecules in the compartment (mmol·L^−1^)
**F0**	Initial concentration of food in the compartment
*A_1_*, *A_2_*, *A_3_*, *A_4_*	Concentrations in molecules A_1_, A_2_, A_3_ and A_4_ in one protocell (mmol·m^−3^)
**A_1,C_, A_2,C_, A_3,C_, A_4,C_**	Concentrations in molecules A_1_, A_2_, A_3_ and A_4_ in the compartment (mmol·m^−3^)
**Fi**	Food input rate in the compartment (mmol·m^−3^·s^−1^)
*P*	Environmental parameter (no unit)
**Pt**	Period of *P* oscillations (s)
**Pa**	Amplitude of *P* oscillations (no unit)
**Df**	Constant for food diffusion between protocells and the environment (mmol·s^−1^·m^−2^)
*V*	Volume of a protocell (m^3^)
**D**	Constant for diffusion of molecules A1, …, A4 between protocells and the environment (m·s^−1^)
*U_1_*, *U_2_*, *U_3_*, *U_4_*	Rates of active intake molecules A1, …, A4 by protocells (mmol·s^−1^·m^−2^)
**fm**	Constant for the food required for protocell maintenance (mmol·m^−3^·s^−1^)
**fu**	Constant for the food required for active intake of molecules A1, …, A4 (m^2^)
**Gr**	Constant for protocell growth ((m^3^·mmol^−1^)^3^)
**L**	Constant for protocell death probability (mmol^−1^)
**S**	Standard deviation of the distribution of change of *U_1_*, …, *U_4_* between generations (mmol·s^−1^·m^−2^)
