# Supplementary material for: Environmental Stability and Its Importance for the Emergence of Darwinian Evolution
Source: Life (Basel). 2023 Sep 25;13(10):1960. doi: 10.3390/life13101960 (PMC10608181; doi:10.3390/life13101960)
Supplement: Supplementary file 1 [file life-13-01960-s001.zip › life-2597880-supplementary.pdf]

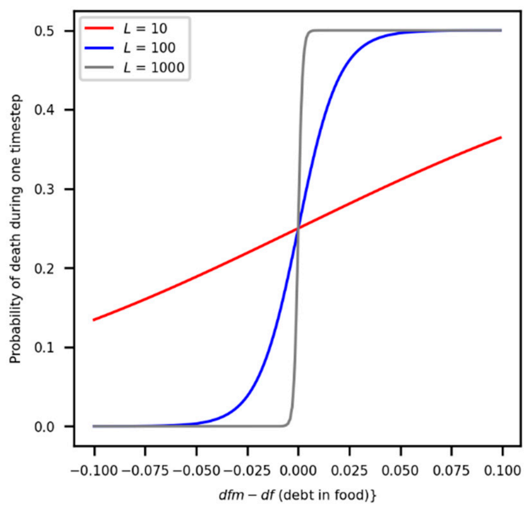

Figure S1: Probability of protocell death depending on food debt

```
import numpy as np

import matplotlib.pyplot as plt


#Number of timesteps:

T = 150


# Initial P

Pi = 100



Pa = 75

Pt = 15

S = 5



   # function describing the evolution of P during the T timesteps:


   x = np.arange(0,T)

   y = Pa*np.sin(2*np.pi*x/Pt)+Pi
```

#Initial food amount in compartment

F = 1

rate of food input in compartment

Fi = 0.2

Concentration of molecules A1, ..., A4 in compartment

Axc = 100

initial number of protocells:

N0 = 100

Type of protocells and initial conditions (alive/dead)

Type1 = np.hstack([np.ones(int(np.floor(N0/2))),np.zeros(int(np.floor(N0/2)))])

Type2 = np.hstack([np.zeros(int(np.floor(N0/2))),np.ones(int(np.floor(N0/2)))])

#factor for death probability

L = 1000

initial protocell volumes

V = 1*np.ones(N0);

volume threshold for division

VT = 2

factor for volume growth of protocells

Gr = 0.005

initial concentration of molecules A1, ..., A4 *in protocells*. To simplify, all these molecules have the same initial oncentrations A here.

A = 100*np.ones(N0)

```
# factor of diffusion for food
DF = 0.01


# factor for food/energy conversion
Fu = 1*10**(-5)

# factor for maintenance energy
Fm = 0.001

# Factor of diffusion for molecules A1, ..., A4.
D = 0.1

# initial rate of active uptake of molecule A, ..., A4.
U = 10*np.ones(N0)

# matrix containing all the info on all protocells. Each protocell corresponds to a line.
# columns in the matrix: protocell volume | 4 columns with A concentrations | 4 columns with rates of active A intake | State and type of the protocell (alive/dead) |
protocells = np.array([V,A,A,A, A, U, U, U, U, Type1, Type2]).T



for t in range(T):


    # at each timestep, food input in the compartment
    F = F+ Fi
```

```
        Fc_tot = 0
        # value of P on timestep t
        P = y[t]
# Initializing the matrix that will contain the daughter protocells. It has the same structure than the matrix 'Cells'.
        daughter = np.empty((0,11))


        #for each active protocell:
        for i in range(Cells.shape[0]):
            # if the protocell is active:
          if protocells[i,9] + protocells[i,10] > 0:
          # protocellular division and stochastic variations ("mutations") if volume superior to VT:
            if protocells[i,0] > VT:
              # defining the value of stochastic variation; it depends on the protocell type.
              if protocells[i,-2] > 0:
                stochC = stochC1;
              if protocells[i,-1] > 0:
                stochC = S;
# defining the mutations of the values of active uptake U1, ..., U4 for daughter protocells
              mutations1 = list();
              for j in range(5,9):
                X = np.random.normal(loc = 0, scale = stochC)
                if X < -Cells[i,j]:
                  X = -Cells[i,j]
                mutations1.append(X)
              mutations2 = list();
              for j in range(5,9):
                X = np.random.normal(loc = 0, scale = stochC)
                if X < -Cells[i,j]:
                  X = -Cells[i,j]
```

```
            mutations2.append(X)

          # defining the characteristics of the two daughter protocells. V = half the one of the
mother protocell, same A concentrations, rates of active A uptakes with stochastic variation, same
type.

          daughter = np.vstack([daughter,np.matrix([[Cells[i,0]/2,

                                   protocells[i,1],Cells[i,2],Cells[i,3],Cells[i,4],

protocells[i,5]+mutations1[0],Cells[i,6]+mutations1[1],Cells[i,7]+mutations1[2],Cells[i,8]+mutations1[
3],Cells[i,-2],Cells[i,-1]],

                                   [Cells[i,0]/2,

                                    protocells[i,1],Cells[i,2],Cells[i,3],Cells[i,4],

protocells[i,5]+mutations2[0],Cells[i,6]+mutations2[1],Cells[i,7]+mutations2[2],Cells[i,8]+mutations2[
3],Cells[i,-2],Cells[i,-1]]])]);

          # mother protocell disappears

          protocells[i,-2] = 0

          protocells[i,-1] = 0


                # otherwise, if no division:

        else:

          V_i = protocells[i,0]

          # entry of food in the protocell through diffusion:

          Fc = DF*F*V_i**(2/3)

          #adding this amount to the total amount of food consumed by all protocells

          Fc_tot = Fc_tot + Fc

          #change of A1, ..., A4 concentrations in the protocell.

          for j in range(1,5):

            Ai = protocells[i,j]

            protocells[i,j] = Ai + (D*(Axc-Ai)+Cells[i,j+4])/V_i**(1/3)

          # Energy required for maintenance

          F_maint = Fm*V_i

          # Energy required for active uptake of molecules A1, ..., A4 (proportional to sum of U
values)
```

```
            F_U = np.sum(Cells[i,5:9])*Fu

            # if there is not enough energy for maintenance and active intake, the protocell has a
probability to die.

            if np.random.rand() <  0.5*(1-1/(1+np.exp(-L*(Fc-F_maint-F_U)))):

              protocells[i,-1] = 0;

              protocells[i,-2] = 0;

            else:

            # food that remains for growth:

              if F_maint + F_U < Fc:

                F_growth = Fc - F_maint - F_U

            # Influence of P on catalyst molecule

                if P <50:

                  Mol = protocells[i,1]

                if P>=50 and P<100:

                  Mol = protocells[i,2]

                if P>=100 and P<150:

                  Mol = protocells[i,3];

                if P >= 150:

                  Mol = protocells[i,4];

            # Protocell growth

                protocells[i,0] = V_i + Gr*(Mol**2)*F_growth

                  #the molecules A are then diluted due to growth:

                for j in range(1,5):

                  protocells[i,j] = protocells[i,j]*V_i/Cells[i,0]


  ### adding daughter protocells


    protocells = np.vstack([Cells,daughter])


  # computing the amount of food remaining in the compartment at the end of the timestep

    if Fc_tot > F:
```

```
    F = 0
else:
    F = F - Fc_tot
```
